# Pitfalls in the statistical examination and interpretation of the correspondence between physician and patient satisfaction ratings and their relevance for shared decision making research

**DOI:** 10.1186/1471-2288-11-71

**Published:** 2011-05-18

**Authors:** Oliver Hirsch, Heidemarie Keller, Christina Albohn-Kühne, Tanja Krones, Norbert Donner-Banzhoff

**Affiliations:** 1 Department of General Practice/Family Medicine, University of Marburg, Germany

## Abstract

**Background:**

The correspondence of satisfaction ratings between physicians and patients can be assessed on different dimensions. One may examine whether they differ between the two groups or focus on measures of association or agreement. The aim of our study was to evaluate methodological difficulties in calculating the correspondence between patient and physician satisfaction ratings and to show the relevance for shared decision making research.

**Methods:**

We utilised a structured tool for cardiovascular prevention (arriba™) in a pragmatic cluster-randomised controlled trial. Correspondence between patient and physician satisfaction ratings after individual primary care consultations was assessed using the Patient Participation Scale (PPS). We used the Wilcoxon signed-rank test, the marginal homogeneity test, Kendall's tau-b, weighted kappa, percentage of agreement, and the Bland-Altman method to measure differences, associations, and agreement between physicians and patients.

**Results:**

Statistical measures signal large differences between patient and physician satisfaction ratings with more favourable ratings provided by patients and a low correspondence regardless of group allocation. Closer examination of the raw data revealed a high ceiling effect of satisfaction ratings and only slight disagreement regarding the distributions of differences between physicians' and patients' ratings.

**Conclusions:**

Traditional statistical measures of association and agreement are not able to capture a clinically relevant appreciation of the physician-patient relationship by both parties in skewed satisfaction ratings. Only the Bland-Altman method for assessing agreement augmented by bar charts of differences was able to indicate this.

**Trial registration:**

ISRCTN: ISRCT71348772

## Background

The correspondence of satisfaction ratings between physicians and patients can be assessed on different dimensions. One may examine whether they differ between the two groups or focus on measures of association or agreement.

Wirtz and Caspar mention several measures to assess interobserver agreement [1]. If the focus is on the differences between two raters, one may use the Wilcoxon signed-rank matched-pairs test. The McNemar test and the marginal homogeneity test test the null hypothesis that the patterns of row and column marginal totals in a contingency table are symmetrical [2,3]. This emphasizes the comparison of the distributions.

One may further use measures of association like the Pearson correlation coefficient, the intra-class correlation coefficient, the Spearman rank correlation coefficient, or Kendall's tau-coefficients depending on the scale of measurement. Weng [4] states that studies have found low correlations between physicians' self-ratings of their performance and the ratings of this performance by evaluators like patients, nurses, or peers. She suggests that physician ratings of the patient-physician relationship may largely be influenced by their patients' symptoms, their functional status, and their prognosis. Using visual analogue scales, Zandbelt et al. [5] revealed patients had a higher overall satisfaction with the encounter when compared to their physicians. The correlation of patients' and physicians' overall satisfaction was significant, but rather small (r = .28). This is confirmed by a study of Bjertness et al. [6], who also found a higher satisfaction of patients with their treatment in a mental health outpatient clinic compared to their physicians. The correlation between the satisfaction ratings of the two groups was r = .37, but patients' ratings showed restriction of range. The variance of the satisfaction ratings in the study of Weng [4] was also quite small, so that the reported correlations between patient and physician satisfaction ratings around r = .4 might be an underestimation [7].

In contrast to the aforementioned approaches, the Bland-Altman method focuses on the agreement between two raters or methods. Bland and Altman state that the use of correlation coefficients in the case of agreement is misleading [8-10]. A correlation measures the strength of a relation between variables but not the agreement. A graphical procedure is proposed by plotting the mean of two methods or ratings against their differences [11]. As a result, it is possible to evaluate the size of the differences, their direction, and their distribution over the range of measurement. The method also supplies the calculation of limits of agreement and their confidence intervals. One then has to decide whether the limits of agreement and the graphical display signal an acceptable level of agreement. The Bland-Altman method for assessing agreement does not deliver p-values and consequently demands an interpretation of the results with regard to the content of the underlying theoretical construct.

Little is known about the correspondence between physician and patient satisfaction regarding a particular treatment or encounter. Only a few studies have addressed the question of how closely satisfaction corresponds between patients and physicians [12]. In the context of shared decision making (SDM) there is a closer relationship between physician and patient. Therefore, it makes sense to ask both co-creators of communication and decision making to evaluate this process. The resulting data is typically characterised by a small number of items, a small number of levels, and a high skewness. Nevertheless, it is an advantage to have the same measure for patients and physicians to directly capture their respective perception of the process of shared decision making.

Controversy still exists regarding how to measure SDM. Some instruments were found to be insufficiently precise to accurately measure this aspect of communication in patient-physician interactions [13,14]. Satisfaction ratings are often used in SDM research to measure the postulated advantages of this approach [15], but they have not been thoroughly examined methodologically, especially the correspondence between patient and physician ratings.

We have found only one study in which the Bland-Altman method was applied to data in the area of shared decision making. Weiss and Peters [16] compared the OPTION scale and the Informed Decision Making Instrument in consultations in general practice. The limits of agreement were quite wide, resulting in an unacceptably low level of agreement which illustrates the aforementioned difficulties in measuring SDM. We have not found studies in this area that compared patient and physician satisfaction ratings with the methods previously stated.

The aim of our analyses was to evaluate methodological difficulties in calculating the correspondence between patient and physician satisfaction ratings and to show the relevance for shared decision making research. Luiz and Szklo [17] advocate the use of more than one statistical strategy to assess interobserver agreement. We followed this reasoning in our study by applying several different approaches to measure association and agreement between physicians and patients.

## Methods

Because of the aforementioned relevance for SDM research, data from an SDM trial are predestined for such analyses. We therefore present data from our randomized controlled trial. The primary aim of this study was to evaluate the effects of a structured tool for cardiovascular prevention (arriba™) on satisfaction level of both patients and physicians in a reciprocal relationship of shared decision making contrasted to the results of a control group with usual care. The primary outcome measure was the Patient Participation Scale (PPS) of which a physician version was constructed. In this paper we present results of secondary analyses on the correspondence between patient and physician satisfaction ratings. The rationale of the trial and its design have been described in detail elsewhere [18,19]. In the intervention group physicians were specially trained to use our shared decision making tool so that their patients were counselled with arriba™. The control group practised usual care. Written informed consent was obtained from the patients and physicians for publication of this report. A copy of the written consent is available for review by the Editor-in-Chief of this journal.

A total of 44 physicians in the intervention group recruited 550 patients, and 47 physicians in the control group recruited 582 patients. We exclusively present the data of the intervention group as the purpose of this paper was to highlight methodological difficulties in calculating the correspondence between patient and physician satisfaction ratings and to show the relevance for shared decision making research. Similar results than those reported were also found in the control group.

Patients' and physicians' satisfaction were measured by two versions of the Patient Participation Scale [20] immediately after index consultation. It consists of six items which can be rated on a Likert scale from one (totally agree) to five (totally disagree) with high scores signifying low participation in and low satisfaction with the consultation (see Appendix).

When analysing the correspondence between physician and patient satisfaction in a primary care setting, one has to remember that patient satisfaction ratings regarding a particular encounter may have a profound ceiling effect [21-23] and are stable over time [24].

There has been a long discussion about whether data from Likert scales are ordinal or metric in nature. Jamieson says that the data from Likert scales is strictly ordinal and should not be analysed with parametric measures [25]. Carifio and Perla are opposed to this view and mention that this ordinalist perspective ignores empirical findings revealing that summations of Likert items can be analysed parametrically. In their opinion, the analysis of single Likert items should only be rarely performed [26]. Even more liberal positions are held by Norman [27], who states that parametric measures are robust so that Likert data generally can be analysed with these measures. Howell [7] even states that "the underlying measurement scale is not crucial in our choice of statistical techniques" (p.9), but he stresses the importance of the interpretation of the obtained results.

Therefore, to explore which method gives the most appropriate interpretation, we applied procedures for different measurement scales, which are implemented in standard statistical software. Regarding the statistical procedures for nominal and ordinal data, we followed the recommendations of the comprehensive approach by Wirtz and Caspar [1]. The authors state that there are no gold standards for the analysis of inter-rater data and advocate the use of several methods. For the evaluation of differences between patients and physicians, we used the Wilcoxon signed-rank matched-pairs test which evaluates whether the median of the differences between two dependent measures in the population is zero [7]. We considered the cluster structure of our data by calculating means of physician and patient satisfaction ratings per physician. In the next step we computed an overall mean and compared patients and their physicians. An effect size for the Wilcoxon test was proposed in the literature, which is calculated by, where Z is the normal approximation of the Wilcoxon test statistic. Cohen considers a cut-off of r = .30 to signal a medium effect [28,29].

The distribution patterns of physicians and patients on the items of the Patient Participation Scale (PPS) were compared using the marginal homogeneity test [30,31]. This examines whether the marginal distributions between raters are systematically different from each other.

We used Kendall's τ-b [7] for associations between patients and physicans. We preferred Kendall's τ-b over Spearman's rank correlation coefficient because it is less sensitive to tied ranks and outliers [32]. With the programme "ComKappa" by Robinson and Bakeman [33] we further calculated weighted kappa coefficients that emphasize the distances between corresponding ratings. Additionally, we calculated the percentage of agreement. We generally considered an α level of .05 as significant.

As an alternative to the aforementioned "traditional" procedures, the parametric Bland-Altman method was applied to measure agreement between physicians and patients [8]. We first computed the differences between the ratings of physicians and patients. A negative difference means that the physician rated an item better than the patient, while a positive rating means that the patient rated an item better than the physician. These differences are then plotted against the average of the single physician and patient ratings. Additionally, lower and upper levels of agreement with their respective 95% confidence intervals are calculated; these must be evaluated regarding their appropriateness with regards to the content of the scale because no significance levels are provided [9-11].

Our general data analysis strategy is in accordance with the recommendations of Donner and Klar regarding the analysis of cluster randomised trials [34]. All statistical analyses were performed with SPSS 17.0, MedCalc 11.2 and ComKappa [33]. We applied Bonferroni correction for multiple testing.

## Results

### Marginal homogeneity test

After crosstabulating the corresponding patient and physician ratings on item level, the inspection of the contingency tables revealed that the categories "neither nor", "disagree", and "totally disagree" were rarely used. We therefore summarised these ratings into one category. After inspecting the contingency tables for each item, it became obvious that patients and physicians mostly differ in their ratings on the first two categories, "strongly agree" and "agree". The physicians in our study were slightly less satisfied than their patients because more physicians rated "agree" when their patients rated "strongly agree" and vice versa. As an example table 1 depicts this asymmetry for item 1 in the intervention group ("My doctor helped me to understand all of the information" versus "I helped my patient to understand all of the information.").

**Table 1:** Contingency table of ratings on PPS item 1 by physicians and patients in the intervention group.

		patients					total
		**strongly agree**	**agree**	**neither nor**	**disagree**	**strongly disagree**	

**physicians**	**strongly agree**	264	21	4	0	0	289
		52.1%	4.1%	0.8%	0%	0%	57.0%
	
	agree	184	16	3	1	1	205
		36.4%	3.2%	0.5%	0.2%	0.2%	40.5%
	
	neither nor	5	1	0	0	0	6
		1.0%	0.2%	0%	0%	0%	1.2%
	
	disagree	5	0	1	0	0	6
		1.0%	0%	0.2%	0%	0%	1.2%
	
	strongly disagree	0	0	0	0	0	0
		0%	0%	0%	0%	0%	0%

**total**		458	38	8	1	1	506
		90.5%	7.5%	1.6%	0.2%	0.2%	100%

Next, we compared the distributional patterns between both groups with marginal homogeneity tests. Results signal significant differences on all items between physicians and patients (table 2), which means that patients and physicians differ in their satisfaction ratings with better ratings by the patients.

**Table 2:** Comparison of the distributions of physician and patient satisfaction ratings in the intervention group with the marginal homogeneity test

Item	marginal homogeneity test (stand. MH)
1	10.47 (p < .001)
2	11.55 (p < .001)
3	11.74 (p < .001)
4	10.80 (p < .001)
5	9.56 (p < .001)
6	11.67 (p < .001)

We applied Bonferroni correction for multiple testing (α = .05/6 = .008).

### Wilcoxon signed-ranks matched-pairs test

We examined differences between patient and physician ratings on item level. The means for each item of the PPS are shown in table 3.

**Table 3:** Comparison of physician and patient satisfaction ratings in the intervention group with the Wilcoxon signed-rank test

	patients	physicians			
**Item**	**mean (sd)**	**mean (sd)**	**mean (sd) of differences**	**median of differences**	**Wilcoxon-test (Z)**

1	1.13 (.14)	1.49 (.39)	0.34 (0.70)	0	-4.56 (p < .001)
2	1.15 (.18)	1.57 (.46)	0.42 (0.77)	0	-4.83 (p < .001)
3	1.09 (.12)	1.46 (.43)	0.37 (0.65)	0	-4.71 (p < .001)
4	1.15 (.20)	1.56 (.43)	0.40 (0.82)	0	-4.21 (p < .001)
5	1.24 (.25)	1.66 (.45)	0.40 (1.00)	0	-4.37 (p < .001)
6	1.12 (.13)	1.59 (.42)	0.46 (0.85)	0	-5.25 (p < .001)

We applied Bonferroni correction for multiple testing (α = .05/6 = .008). Item means and standard deviations and means, standard deviations, and medians of the differences are listed. The scale ranges from 1 (totally agree) to 5 (totally disagree).

It is apparent that all means of the scale values lie between one and two with small standard deviations, especially for patients. This signals that our satisfaction ratings are skewed with an overrepresentation of positive ratings. This is further supported by considering the fact that the means of the differences are smaller than their respective standard deviations. Using the non-parametric Wilcoxon signed-rank test, significant differences occurred on all items of the PPS after Bonferroni correction for multiple testing, although the medians of the differences on all items are zero. Generally, patients were more satisfied than physicians. The effect sizes for the Wilcoxon test range from r = .64 to r = .79 and can be considered large [29].

### Weighted kappa and Kendall's tau-b

We then calculated the association between patient and physician ratings on the PPS by using Kendall's τ-b and weighted kappa coefficients [1,7]. Both coefficients are generally low (<.10), showing no significant associations (table 4).

**Table 4:** Association between physician and patient satisfaction ratings measured with weighted kappa, percentage of agreement, and Kendall's τ-b

Item	weighted kappa	percentage of agreement	Kendall's τ-b
1	.01 (p = .71)	55.3	.04 (p = .38)
2	.03 (p = .34)	51.3	.09 (p = .04)
3	.03 (p = .34)	58.3	.08 (p = .09)
4	.01 (p = .71)	49.5	-.001 (p = .99)
5	.01 (p = .71)	47.1	.08 (p = .09)
6	.04 (p = .27)	52.8	.09 (p = .05)

We applied Bonferroni correction for multiple testing (α = .05/6 = .008).

The percentages of agreement are also low. For example, in item 1 the percentage of agreement is only 55.3%. In table 1, one can see that another 36.4% of the cross tabulated ratings indicate that the physicians rated "agree" and the respective patients rated "strongly agree".

### Bland-Altman method of agreement

Table 5 depicts the results of the Bland-Altman method of agreement in the intervention group. We take item 1 as an example to illustrate the data. The lower limit of agreement of -1.02 (95% CI: -1.12 to -0.92) means that 95% of differences that signal better ratings of the physicians lie within about one scale point. The upper limit of agreement of +1.71 (95% CI: +1.60 to +1.81) means that 95% of differences that signal better ratings of the patients lie within about 1.7 scale points. Referring to the five point scale of the PPS, the lower limit of agreement is small while the upper limit of agreement is too wide.

**Table 5:** Lower and upper limits of the Bland-Altman method for assessing agreement between physicians and patients in the intervention group and percentages of differences within these limits and within +- 1 scale point

	lower limit (95% confidence interval)	upper limit (95% confidence interval)	percentage of differences within limits	differences -1 to +1 point
Item 1	-1.02 (-1.12 to -0.92)	+1.71 (+1.60 to +1.81)	96.8%	96.8%

Item 2	-1.09 (-1.20 to -0.97)	+1.93 (+1.81 to +2.04)	97.6%	94.0%

Item 3	-0.92 (-1.01 to -0.82)	+1.65 (+1.55 to +1.75)	96.6%	97.1%

Item 4	-1.20 (-1.32 to -1.08	+2.00 (+1.87 to +2.12)	96.4%	93.4%

Item 5	-1.56 (-1.71 to -1.40)	+2.36 (+2.20 to +2.51)	93.7%	87.8%

Item 6	-1.21 (-1.34 to -1.08)	+2.13 (+2.00 to +2.26)	95.5%	92.6%

The scale ranges from 1 (totally agree) to 5 (totally disagree).

The upper limits signal larger differences in the sense of an overrepresentation of better patient satisfaction ratings. 96.8% of the differences on item 1 are within the limits of agreement and 96.8% of the differences are also within plus/minus one scale point. The high percentages of differences in the range of -1, 0 or +1 scale point make clear that the ratings of physicians and patients are quite close with the tendency of physicians to rate a bit worse than the patients. An exception is item 5 (decision about further treatment) with 12.2% of differences outside of the range of -1, 0 or +1 scale point.

**Figure 1:**
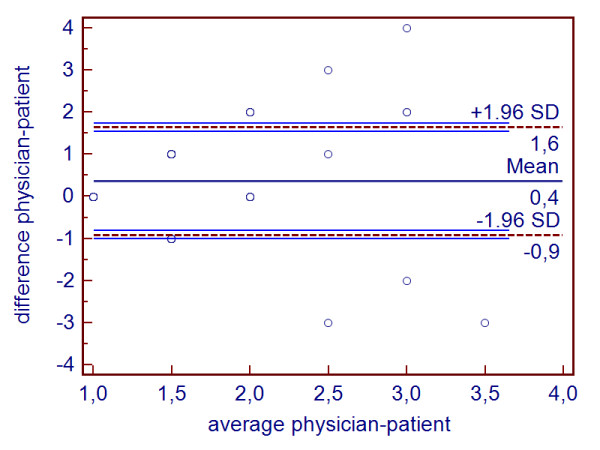
**Bland-Altman plot of item 3 of the PPS in the intervention group**.

In figure 1 we present an example of a Bland-Altman plot.

The trumpet shape of the data points suggest that the differences increase with higher averages. This is misleading because the data points represent very different numbers of observations. Altman and Bland [35] and Smith et al. [36] propose to supplement the Bland-Altman plot with a bar chart of the differences between methods or observers. When the range of observed values is small relative to the number of observations the Bland-Altman method does not seem to be appropriate. Due to the fact that the data points in our example represent different numbers of observations, the Bland-Altman plot does not reveal much about the data distribution.

**Figure 2:**
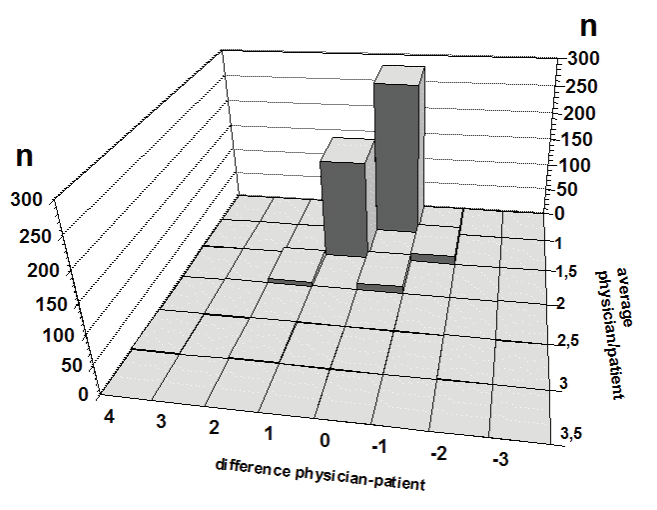
**Three dimensional Bland-Altman plot of item 3 of the PPS in the intervention group**.

Figure 2 further illustrates this issue by translating the Bland-Altman plot into a three dimensional bar chart. There it is immediately obvious that the data points represent very different numbers.

**Figure 3:**
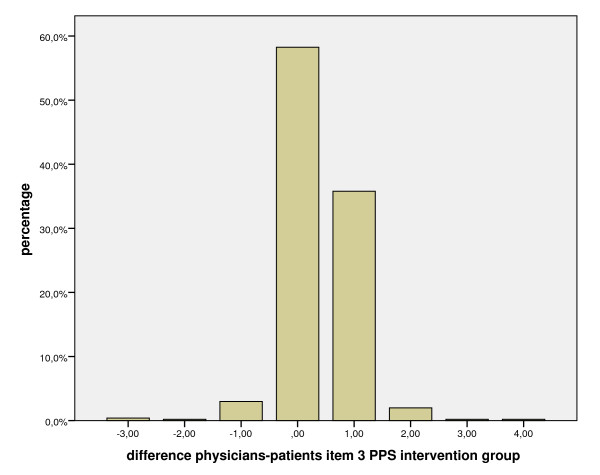
**Bar chart of the differences between physicians and patients on item 3 of the PPS in the intervention group**.

Figure 3 exemplifies the distribution of differences on the same item. It shows that 97.1% of the differences between physicians and patients on item 3 of the PPS ("My doctor answered all of my questions./I answered all of my patient's questions.") are within plus/minus one scale point. There are different proportions above and below a difference of zero. Thirty-five percent of the difference have a value of plus one scale point, which means that physicians rate this item more critically than the patients. Nevertheless this is a high concordance theoretically and clinically.

## Discussion

The aim of our study was to evaluate methodological difficulties in calculating the correspondence between patient and physician satisfaction ratings and to show the relevance for shared decision making research. To our knowledge, ours is the first study that examines this approach in the context of shared decision making from a methodological point of view.

### Differences

We found significant differences between patient and physician ratings on almost all items of the PPS using the Wilcoxon matched-pairs signed-rank test. The means of the differences between patient and physician ratings were smaller than their respective standard deviations and therefore signal a profound skewness of the data [37]. The medians of the differences between patient and physician ratings were zero on all items, which shows that the results of the Wilcoxon matched-pairs signed ranks tests are not appropriate [38]. The medium to large effect sizes are an effect of the large sample size as the square root of the sample size is in the denominator. It is suggested that patients were more satisfied with the shared decision making process than physicians. As the scores of the patients are, in most instances, slightly better than those of the physicians, the resulting significant differences are more or less trivial and tell us nothing about the size of the effect [28].

The distributions in the contingency tables were significantly different between patients and physicians with patients expressing a more positive view. The marginal homogeneity test tests the null hypothesis that the patterns of row and column marginal totals in a contingency table are symmetrical. It ignores the agreement diagonal and therefore is not suitable for detecting differences between raters because it ignores the extent of agreement. Consequently, an unusually high priority is given to disagreement. It is also debated whether this test captures the ordinal nature of rating data [2,3].

After a closer inspection of the raw data, it is obvious that the patients more often use the category "strongly agree" on the PPS when their physicians choose the category "agree". The remaining three categories were rarely used. The significant differences of the distributions in the contingency tables can also be explained by the known fact that such measures like the marginal homogeneity test are sample size dependent. In a large sample like ours, even relatively small numerical differences reach statistical significance [7,39].

### Associations

We found low coefficients of association between patient and physician ratings. Hence, one could argue that patient and physician satisfaction are very much different and have a low correspondence. However, the low coefficients of association between patient and physician satisfaction can be explained by restriction of range and the skewness of our data. This is confirmed by the meta-analysis of Hall and Dornan [23]. They demonstrated that the small magnitudes of many correlates of satisfaction could be largely due to restriction of range in the satisfaction measures.

We consider weighted kappa to be more a coefficient of association; this is in agreement with Graham and Jackson [40], who also question the use of weighted kappa as an index of agreement. According to them, weighted kappa is more a measure of association that produces counterintuitive results under certain circumstances (e.g., high values at low level of agreement). Ludbrook [2] also shares this opinion and demonstrates that weighted kappa is not able to unravel a systematic bias between observers.

In their study of the inter-rater reliability of the Frenchay Activities Index in stroke patients, Post and de Witte [41] also reported a low weighted kappa in the context of a high percentage agreement due to a skewed distribution of scores. This paradoxical finding is confirmed by Booth et al. [42], Donker et al. [43], and by Ovre et al. [44]. Ahlén et al. [45] validated a questionnaire to assess physician-patient agreement at the consultation. The authors found low kappa coefficients, but high agreement regarding the index of validity and the indices of proportional agreement. This discrepancy may occur when there are high numbers of agreement and low numbers of non-agreement between observers and when the marginal totals in a fourfold contingency table are not balanced [46]. We could not apply the proposed indices of proportional agreement in our study because the necessary dichotomization of the items on the patient participation scale was not reasonable with regards to the content [47,48]. We also conclude that the kappa coefficient only leads to interpretable results when the distribution over the respective categories is quite uniform. The percentages of agreement are also misleading in that they signal a low agreement, although the difference between physicians rating "agree" and their respective patients rating "strongly agree" is not of much clinical relevance. A class of models to further describe rater agreement is proposed by Agresti [49].

In the case of skewed satisfaction ratings in the context of shared decision making, methods of analysis based on hypothesis testing and global indices are obviously misleading. Agreement has to be quantified and judged with regards to content of the underlying construct [9]. A correlation depends on the range and distribution of the variables and does not incorporate a possible bias between these variables. It measures the degree of association, but not agreement [10]. In their systematic literature review, Schmidt and Steindorf [50] also criticize that the use of correlation coefficients in questionnaire validation studies leads to misleading conclusions. Measures of association depend on the variance or the prevalence of the operationalised construct in the respective sample. They plead for the application of the Bland-Altman method as the preferable measure for questionnaire evaluations.

### Agreement

The Bland-Altman method augmented by bar charts of differences between physician and patient ratings was the best measure to capture the theoretically and clinically relevant high agreement regarding satisfaction with the encounter. It does not involve statistical testing to evaluate chance and consequently demands an interpretation of the results with regards to the content of the underlying theoretical construct. This might be seen as a disadvantage, however, we consider this to be an advantage because researchers often rely on frequently questionable p-values. Murphy et al. [39] emphasize that traditional tests of significance do not directly assess the size or importance of effects. In large samples, even negligible effects are statistically significant. This fact demonstrates the importance of consulting appropriate effect size measures to evaluate the size of the effect [28]. A small effect, may nevertheless, be clinically important in a certain area. This stresses the importance of an interpretation with regards to content and not just focusing on p-values.

The calculation of the 95% limits of agreement is grounded on the assumption that the differences between two methods or raters are normally distributed. In our study, the distributions of satisfaction ratings are skewed. Nevertheless, Bland and Altman state that a non-normal distribution of differences may not be a serious violation [9]. Our data reveals that approximately 95% of the differences between physicians and patients on all items of the PPS are indeed within two standard deviations of the mean.

The relatively high standard deviations of differences in our data pose a problem for the Bland-Altman method. The resulting higher limits of agreement signal less agreement than is actually the case when inspecting the bar charts of differences between physicians and patients. In spite of this Bland and Altman do not recommend excluding outliers [9]. As previously mentioned, there is a long discussion about the parametric analysis of Likert scale data [25,26]. The results of the Bland-Altman method, supplemented by bar charts of differences provide the most appropriate interpretation of our correspondence data. Smith et al. [36] argue for the use of the Bland-Altman method even when the data has few categorical values. According to the authors, supplementing the method with bar charts makes it capable of effectively analysing agreement data even when the number of unique values is limited. Schmidt and Steindorf [50] showed that the Bland-Altman method is adequate and robust for questionnaire data. The method was able to detect serious bias in questionnaire data which was undetected by correlation coefficients. Numerous studies have applied the Bland-Altman method to questionnaire data on scale and item level and to data with limited ranges [16,51-57]. We therefore consider our argumentation to be supported by the view of those authors who state that Likert scale data can be analysed this way [7,27].

Twomey and Viljoen propose to use the Bland-Altman method instead of the Wilcoxon matched-pairs signed ranks test [38]. Smith et al. [36] prefer the Bland-Altman method over weighted kappa because of easier interpretation of the scale of measurement and the greater insight through graphical presentation.

## Conclusions

We illustrated the difficulty of finding an appropriate method for the analysis of skewed satisfaction data in shared decision making. None of the presented methods was fully able to satisfactorily capture the theoretically and clinically relevant agreement between physicians and patients that was shown in simple cross tabulations. Only the Bland-Altman method, augmented by bar charts of differences between physicians and patients, revealed a higher agreement than was proposed by other methods.

We recommend closely inspecting basic graphical representations of agreement data because traditional statistical measures can produce misleading results in this area. Our data revealed that what visually appears to be a fairly good agreement might produce high differences and low levels of association. This finding is relevant for research in SDM because satisfaction ratings with the aforementioned properties are especially used in this area.

## Appendix

Patient Participation Scale (PPS): patient and physician version

1. My doctor helped me to understand all of the information./I helped my patient to understand all of the information.

2. My doctor understood what is important for me./I understood what is important for my patient.

3. My doctor answered all of my questions./I answered all of my patient's questions.

4. I was sufficiently involved in decisions about my treatment./I sufficiently involved my patient in decisions about his treatment.

5. I have decided the further treatment together with my doctor and I am satisfied with the result./I have decided the further treatment together with my patient and I am satisfied with the result.

6. I am satisfied with the manner by which my treatment has been discussed and decided./I am satisfied with the manner by which the treatment of my patient has been discussed and decided.

## Competing interests

The authors declare that they have no competing interests.

## Authors' contributions

OH developed the concept for data analysis, performed the statistical analyses, and drafted the manuscript. HK participated in the study design and coordination, the rationale for the data analyses, carried out the study, and helped to draft the manuscript. CAK assisted in developing the concept for data analysis and in performing the statistical analyses. TK participated in the study design and coordination, the rationale for the data analyses, carried out the study, and helped to draft the manuscript. NDB participated in the study design and coordination, the rationale for the data analyses, and helped to draft the manuscript. All authors read and approved the final manuscript.

## Pre-publication history

The pre-publication history for this paper can be accessed here:

http://www.biomedcentral.com/1471-2288/11/71/prepub
